# Chronic Phase Shifts of the Photoperiod throughout Pregnancy Programs Glucose Intolerance and Insulin Resistance in the Rat

**DOI:** 10.1371/journal.pone.0018504

**Published:** 2011-04-06

**Authors:** Tamara J. Varcoe, Nicole Wight, Athena Voultsios, Mark D. Salkeld, David J. Kennaway

**Affiliations:** Research Centre for Reproductive Health, Robinson Institute, University of Adelaide, Adelaide, South Australia, Australia; Vanderbilt University, United States of America

## Abstract

Shift work during pregnancy is associated with an increased risk for preterm birth and low birth weight. However, the impact upon the long term health of the children is currently unknown. In this study, we used an animal model to determine the consequences of maternal shift work exposure on the health of the adult offspring. Pregnant rats were exposed to chronic phase shifts (CPS) in their photoperiod every 3–4 days throughout gestation and the first week after birth. Adult offspring were assessed for a range of metabolic, endocrine, circadian and neurobehavioural parameters. At 3 months of age, male pups exposed to the CPS schedule *in utero* had increased adiposity (+29%) and hyperleptinaemia (+99% at 0700h). By 12 months of age, both male and female rats displayed hyperleptinaemia (+26% and +41% respectively) and hyperinsulinaemia (+110% and +83% respectively). 12 month old female CPS rats displayed poor glucose tolerance (+18%) and increased insulin secretion (+29%) in response to an intraperitoneal glucose tolerance test. In CPS males the glucose response was unaltered, but the insulin response was reduced by 35%. The glucose response to an insulin tolerance test was decreased by 21% in CPS females but unaltered in males. Disruption of circadian rhythmicity during gestation resulted in gender dependent metabolic consequences for the adult offspring. These results highlight the need for a thorough analysis of shift work exposure *in utero* on the health of the adult offspring in humans.

## Introduction

With the incidence of shift work increasing rapidly in our society, so is our understanding of the consequences of these working schedules upon health and wellbeing. Shift workers are at increased risk of developing many chronic diseases including coronary heart disease, gastric ulcers, diabetes and other metabolic disturbances [1]. There is also epidemiological evidence associating shift work and night work during pregnancy with preterm birth, low birth weight, and spontaneous abortion [2], [3], [4], [5], [6]. The mechanisms responsible for these poor health outcomes are not understood, but given that shift work schedules disrupt hormonal and sleep rhythmicity, eating patterns and light exposure [7], it is conceivable that abnormal rhythmicity may be an important causal factor.

Circadian rhythms are established within the suprachiasmatic nucleus (SCN) of the hypothalamus. Information about the photic environment is received through the retina and transmitted to the SCN. It is here that a molecular feedback loop involving at least 9 core clock genes is responsible for the establishment and maintenance of circadian rhythms [8]. This information is then transmitted via neural or hormonal routes (e.g. melatonin) to the rest of the body. Peripheral tissues also express core clock genes, as well as a number of tissue specific regulatory and effector genes, in a rhythmic manner. Through this mechanism, timing of sleep, arousal, metabolism, cardiovascular function and reproduction are timed to occur at very specific and appropriate times of day. However, for shift workers the timing of these physiological parameters becomes disrupted. Behaviours such as sleep, activity and food consumption are all shifted to match the work schedule, but the majority of shift workers do not correspondingly adjust their body's central SCN rhythmicity [7]. This means there is a mismatch between central circadian function and the enforced schedules.

Over the past decade a considerable body of evidence has emerged showing that circumstances during gestation may have lifelong programming effects on the offspring in both humans and animal models. Factors such as poor maternal nutrition, prenatal stress and exposure to medicinal and social drugs have all been demonstrated to have negative health consequences for the offspring that continue into adulthood [9], [10], [11]. It is therefore important to consider the implications of shift work during pregnancy on the health of the offspring throughout development into adults. Consequently, we conducted experiments to gain a better understanding of the impact of shift work during gestation on the long term health of the offspring. To achieve this we utilised a protocol whereby rats were exposed to repeated phase shifts in the photoperiod throughout gestation and for 1 week after birth, thereby significantly disrupting rhythmicity during this period. The impact of this schedule on pregnancy outcomes, growth, metabolic health, neurobehavioural development and circadian rhythm function was assessed in the adult offspring.

## Methods

### Animals

**Figure 1:**
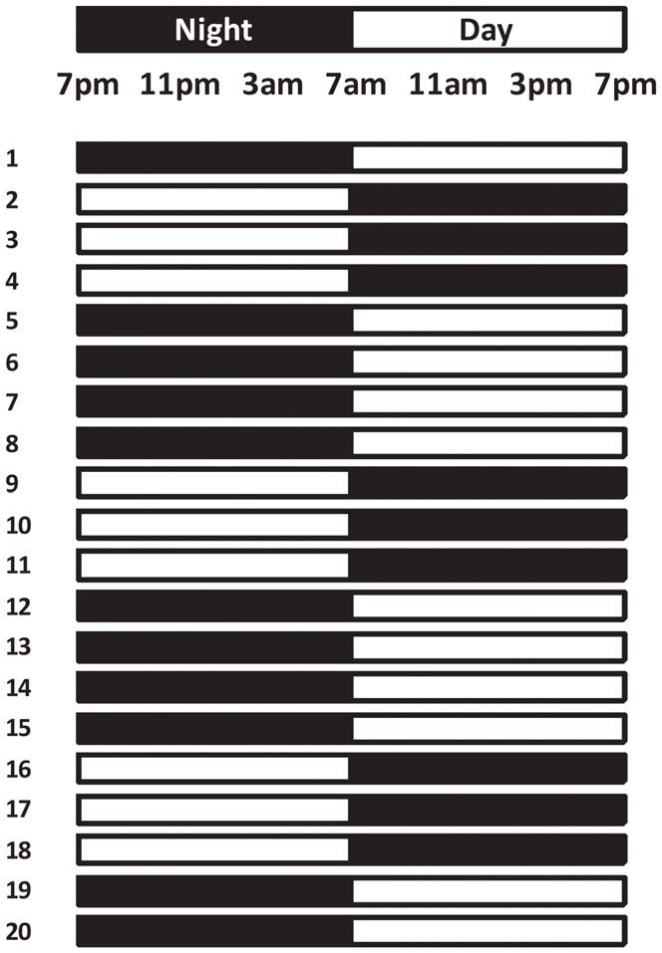
Schematic of the CPS protocol used throughout gestation and for 1 week after birth. Animals were exposed to changes in the timing of darkness/sleep opportunities similar to that experienced by rotating shift workers. Animals had their photoperiod shifted by 12 hours for 3 consecutive cycles, after which the dark phase was extended for 12 hours to restore the original photoperiod for a further 4 days.

**Figure 2:**
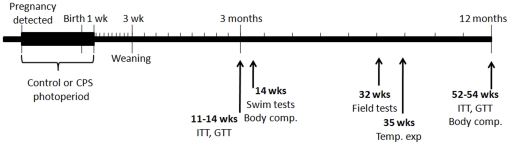
Timeline for assessment of health parameters in CPS and control offspring.

All experiments were approved by the University of Adelaide Animal Ethics Committee and were conducted in accordance with the Australian Code of Practice for the Care and Use of Animals for Scientific Purposes. Mature albino wistar female rats were housed with males (2:1) until pregnancy was confirmed by vaginal smears for sperm. Upon detection of mating, females were maintained on either control lighting conditions (12 light:12 dark, lights on at 0700h) or exposed to repeated phase shifts throughout gestation and for 1 week after birth. The chronic phase shift (CPS) protocol involved manipulating the lighting schedule so that every 3–4 days the photoperiod was completely reversed (Figure 1). All offspring were then maintained on control lighting conditions up to the age of 12 months. Control and CPS offspring were assessed for a range of metabolic, circadian and neurobehavioural measures at different ages (Figure 2).

### Pregnancy Outcomes

Gestation length, litter size and birth weight was recorded. Following birth, all pups were weighed every second day until weaning. Subsets of both male and female rats were weighed every 4 weeks until 12 months of age (n = 15 per group, per gender).

### Metabolic Function

At 3 months of age male CPS and control offspring were killed by decapitation at 0700, 1100, 1500, 1900, 2300 and 0300h (n = 6 per treatment). Major organs including brain, heart, liver, kidney, gastrocnemius muscle, epigonadal fat, retroperitoneal fat, spleen, pancreas, uterus/testes and adrenals were rapidly dissected and weighed, and trunk blood collected into lithium-heparin tubes. At 12 months of age a separate cohort of control and CPS offspring of both genders were killed by decapitation at 4 hourly intervals over 24 hours (n = 2–4 per time point, per gender), with trunk blood collected and tissues dissected and weighed. Plasma was separated with glucose assayed by colorimetric enzymatic analysis on a Hitachi automated centrifugal analyzer with the use of kits from Roche Diagnostic. Insulin and leptin was assayed by RIA using kits obtained from Linco Research (St. Charles, MO).

At 3 and 12 months of age a separate cohort of male and female rats were subjected to intraperitoneal glucose tolerance (IPGTT) and insulin tolerance tests (IPITT). For the IPGTT, overnight fasted animals were injected intraperitoneal with glucose (1 g/kg body wt; Sigma, St. Louis, MO) 2–3 hours after lights on. Blood was obtained from the tail vein before and 15, 30, 60, 90 and 120 min after glucose administration. Glucose was analyzed by the dehydrogenase method (HemoCue, Angelholm, Sweden), with additional blood collected into lithium-heparin microvettes at all time points for subsequent insulin assay. For the IPITT, food was withdrawn at the beginning of the test and the rats were injected intraperitoneally with insulin (0.75 IU/kg body wt; Actrapid) 2–3 hours after lights on. Blood (5 µl) was obtained from the tail vein before and 5, 10, 15, 30, 60, 90 and 120 minutes after insulin administration for glucose determination. The area under the glucose and insulin curves obtained from the IPGTT was calculated using GraphPad Prism (version 5.01 for Windows, GraphPad Software, San Diego, California, USA). Each value is the area relative to the time  = 0 value for each animal and represents the change of either glucose or insulin from baseline. For the IPITT, the area above the glucose curve was calculated. Each value represents the area relative to the time  = 0 value for each animal and represents the change of glucose from baseline.

### Circadian Function

At 35 weeks of age core body temperature was continuously monitored every 10 minutes in a subset of male rats using Thermochron iButton® DS1291H (Dallas Maxin) inserted into the peritoneal cavity. Following 2 days in 12L:12D, the rats were maintained in constant darkness and either exposed to a single 15 minute, 300 lux light pulse 4 or 10 hours after the onset of subjective darkness (2300h or 0500h respectively). After 1 week the animals were killed, the temperature data retrieved from the iButtons, and the time of the morning temperature decline used as a primary phase marker for each rat. The offset was defined as the time at which the temperature first decreased below the mean temperature for that animal and remained below this threshold for at least 30 minutes.

### Neurobehavioural Development

Behavioural despair and anxiety were assessed in a separate cohort of 14 week and 32 week old male rats respectively. Anxiety was assessed using an open field test, and behavioural despair using the forced swim test. The open field test was conducted in a brightly lit square arena (100 cm ×100 cm) surrounded by 30 cm high walls, with a video camera mounted above. Animals were placed in the centre and over a 5 minute period the latency (time before all four feet left the centre square), number of outer and inner squares crossed, and the percentage of outer squares crossed compared to the total number of squares crossed was recorded. The forced swim test was conducted in a cylindrical perspex container (height 60 cm, diameter 25 cm). The container was filled with tap water to a level of 35 cm and maintained at 25°C throughout the experiment. Animals were placed in the container for five minutes and videotaped. Using a stopwatch, the time spent immobile over the test period was recorded for each animal. A rat was judged to be immobile when it remained floating, making only the necessary movements to keep its head above water. Data for the open field and forced swim tests were analysed by two independent assessors, blinded to the treatment group.

### Statistics

SPSS v.17 was used for all statistical analyses. Birth weight was analysed using Linear Mixed Models. ANOVA was used to analyse growth with time as a repeated measures factor. Tissue weight, hormonal analyses and area under/above the curves were analysed using ANOVA with litter size as a covariate. Behavioural tests were analysed using non-parametric Mann-Whitney tests. All treatment and control groups utilised animals from diverse litters with never more than 1 animal from each litter per group. One outlier was removed from the 12 month old control male IPITT group due to non-response to insulin administration presumably due to injection failure. The probability value used to identify statistical significance was P<0.05.

## Results

### Pregnancy Outcomes

A total of 22 control and 18 chronic phase shift (CPS) litters were born. There was no significant difference in gestation length (P>0.05, 21.6±0.4 and 22.1±0.2 days, control and CPS respectively), litter size (P>0.05, 12.11±0.6 and 12.0±0.7, control and CPS respectively), survival to weaning (P>0.05, 98.6 and 98.6% control and CPS respectively) or birth weight (P>0.05, 6.55±0.1 and 6.49±0.1 g, control and CPS respectively) between offspring born to CPS or control dams.

### Growth and Development

**Figure 3:**
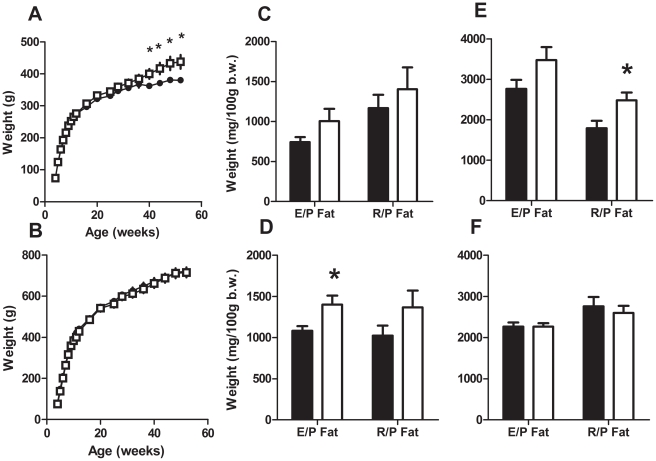
Growth and body composition of adult offspring following exposure to CPS *in utero*. **A–B**: Post weaning growth rate trajectories of female (A) and male (B) CPS (□) and control (•) offspring (n = 15/treatment/group). **C–F**: Epigonadal and retroperitoneal fat weights of 3 month old female (C, n = 8/treatment), 3 month old male (D, n = 8/treatment), 12 month old female (E, n = 14/treatment) and 12 month old male (D, n = 18/treatment) CPS and control offspring. The data are the mean ± SEM, * P<0.05.

The body weights of female offspring of CPS exposed mothers were similar to those born to the controls until 40 weeks of age. After this time the body weights diverged such that by 52 weeks of age they were 15% heavier than the control offspring (Figure 3A). Female CPS offspring at 3 months of age displayed a non-significant trend for increased epigonadal (+36%) and retroperitoneal (+20%) fat weight (Figure 3C). By 12 months of age CPS females displayed significantly increased retroperitoneal fat weight (40%, F_1,27_ = 5.3, P<0.05, Figure 3E).

There was no significant difference in weight between male CPS and control offspring at any age (Figure 3B). Male CPS offspring had significantly greater epigonadal fat deposits (+29%, F_1,13_ = 5.0, P<0.05, Figure 3D) at 3 months of age. By 12 months of age however, there was no difference in either epigonadal or retroperitoneal fat weight between CPS or control male offspring (Figure 3F).

### Metabolic Function

**Figure 4:**
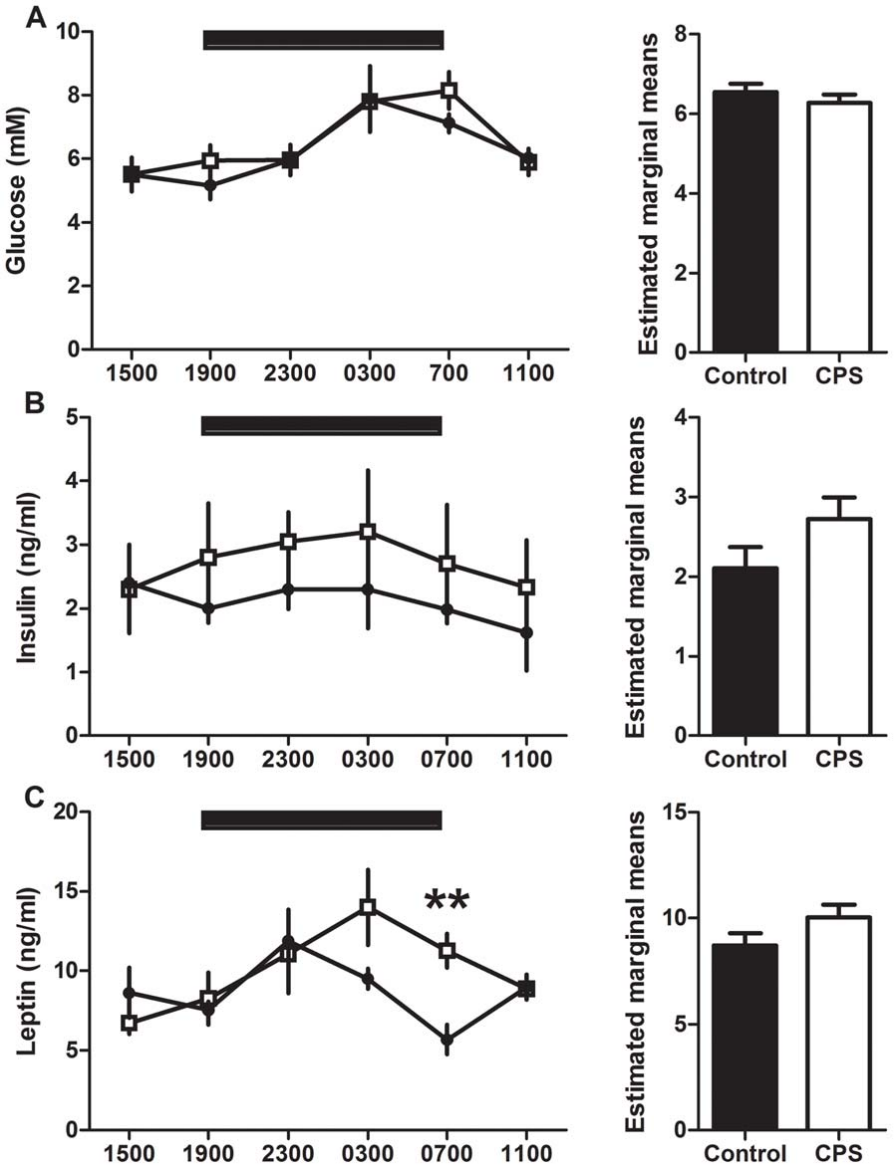
24 hour metabolite analysis of 3 month old offspring following CPS exposure *in utero*. Plasma glucose (A), insulin (B) and leptin (C) concentrations of 3 month old male CPS (□) and control (•) offspring. The data are the mean ± SEM (n = 6/treatment/time point), ** P<0.01.

Plasma glucose did not vary with treatment (F_1,54_ = 0.8, P>0.05) but varied with time of day (F_5,54_ = 7.5, P<0.001) such that peak levels occurred between 0300h and 0700h for CPS and control 3 month old male offspring (Figure 4A). Plasma insulin did not vary with treatment (F_1,59_ = 2.8, P>0.05) or time of day (F_5,59_ = 0.4, P>0.05, Figure 4B). Plasma leptin did not vary with treatment (F_1,59_ = 2.5, P>0.05) but varied with time of day (F_5,59_ = 3.1, P<0.05) such that peak levels of leptin occurred at 2300h and 0300h for control and CPS offspring respectively (Figure 4C). Plasma leptin was higher (+99%) in the CPS rats compared to the controls at 0700h (t_10_ = 4.0, P<0.01).

**Figure 5:**
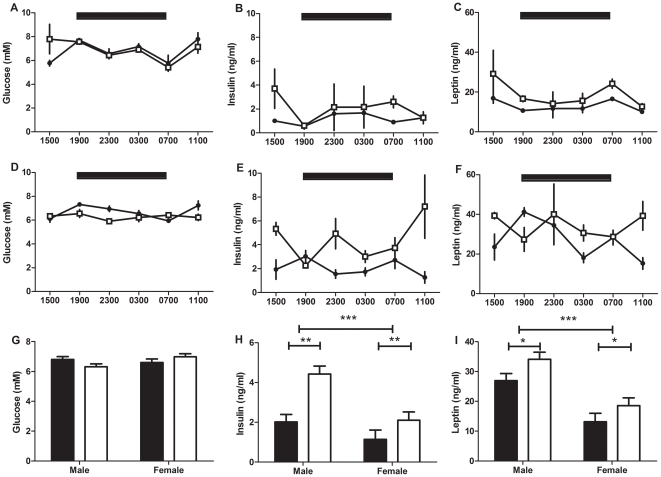
24 hour metabolite analysis of 12 month old offspring following CPS exposure *in utero*. Plasma glucose, insulin and leptin concentrations of 12 month old female (A, B, C) and male (D, E, F) CPS (□) and control (•) offspring. Estimated marginal means of female and male glucose, insulin and leptin concentrations (G, H, I). The data are the mean ± SEM (n = 2–4/treatment/gender/time point),*P<0.05 **P<0.01 ***P<0.001.

Plasma glucose did not vary with treatment (F_1,43_ = 0.1, P>0.05) or time (F_5,43_ = 0.6, P>0.05) in female and male 12 month old rat offspring (Figure 5). Plasma insulin varied with treatment (F_1,44_ = 12.5, P<0.01) in females and males, but not with time of day (F_5,44_ = 1.0, P>0.05, Figure 5). Insulin was increased by 83% and 110% and in female and male rats respectively. Plasma leptin varied with treatment (F_1,42_ = 5.7, P<0.05) but not with time of day (F_5,42_ = 1.2, P>0.05). Leptin was 41% and 26% higher in female and male CPS exposed rats respectively.

**Figure 6:**
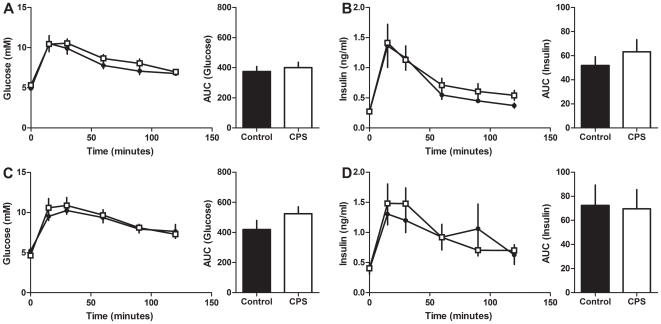
Glucose tolerance in 3 month old CPS and control offspring. Blood glucose and plasma insulin concentrations of CPS and control 3 month old female and male offspring following a 1 g/kg intraperitoneal injection of glucose. A: female blood glucose, B: female insulin, C: male blood glucose, D: male insulin. CPS (□) control (•). The data are the mean ± SEM (n = 7/treatment/gender). Area under the glucose and insulin curves were calculated relative to the baseline value for each rat.

**Figure 7:**
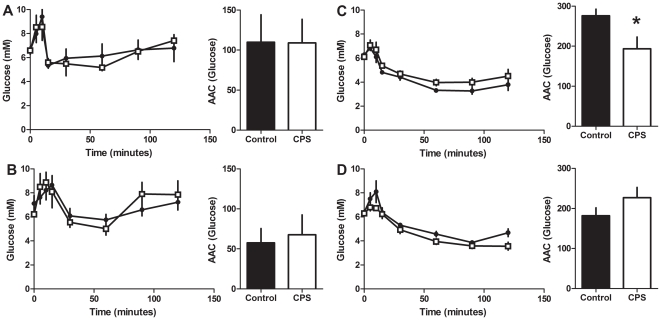
Insulin tolerance in adult CPS and control offspring. Blood glucose concentrations of CPS and control 3 and 12 month old female and male offspring following a 0.75 IU/kg intraperitoneal injection of insulin. A: 3 month old female; B: 3 month old male; C: 12 month old female; D: 12 month old male. CPS (□) control (•). The data are the mean ± SEM (n = 7/treatment/gender), *P<0.05. Area above the glucose curves were calculated relative to the baseline value for each rat.

The plasma glucose response to the IPGTT at 3 months did not vary with treatment in either male or female offspring (Figure 6). Similarly, glucose stimulated insulin secretion did not vary with treatment in either male or female offspring compared to the controls (Figure 6). The plasma glucose response to the IPITT at 3 months did not vary with treatment in either male or female offspring (Figure 7A and 7B).

**Figure 8:**
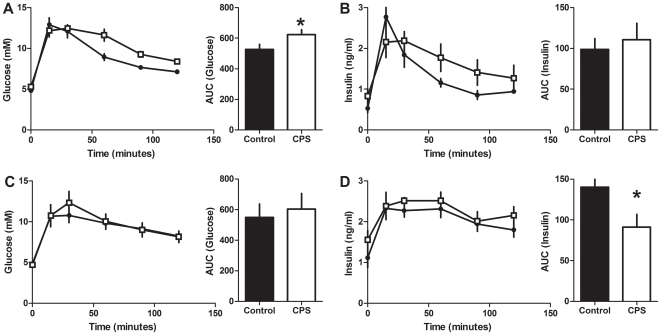
Glucose tolerance in 12 month old CPS and control offspring. Blood glucose and plasma insulin concentrations of CPS and control 12 month old female and male offspring following a 1 g/kg intraperitoneal injection of glucose. A: female blood glucose, B: female insulin, C: male blood glucose, D: male insulin. CPS (□) control (•). The data are the mean ± SEM (n = 7/treatment/gender), *P<0.05. Area under the glucose and insulin curves were calculated relative to the baseline value for each rat.

The glucose response to the IPGTT in 12 month old females varied with treatment (F_1,11_ = 4.3, P<0.05, Figure 8A). This was mainly due to the prolonged elevation (+30%) of glucose between 60 and 120 minutes post injection. The insulin response to the IPGTT did not vary with treatment (F_1,11_ = 0.2, P>0.05, Figure 8B). However, the pattern of insulin secretion was different such that CPS animals had a blunted initial response, followed by elevated insulin levels from 30 to 120 minutes post injection. The insulin response during this second phase varied with treatment (F_2,22_ = 4.5, P<0.05). The glucose response to the IPITT varied with treatment (F_1,11_ = 5.2, P<0.05) such that CPS exposed female rats had a 21% decrease in the area above the glucose curve (Figure 7C).

The plasma glucose response to the IPGTT in males at 12 months of age did not vary with treatment (Figure 8C). However, glucose stimulated insulin secretion varied with treatment (F_1,11_ = 6.6, P<0.05, Figure 8D) such that the area under the insulin curve was 35% lower in the CPS male rats. The glucose response to the IPITT did not vary with treatment (F_1,11_ = 3.2, P>0.05, Figure 7D).

### Circadian Rhythm Function

There was no effect of CPS on the basal, minimum or maximum core body temperature of offspring in 12L:12D or during constant darkness. Nor was there any effect on the phase of the temperature rhythm (Figure S1). The phase delay and advance of the temperature rhythms induced by single light pulses 4 or 10 hours after subjective lights off were similar in offspring of both control and CPS exposed mothers (Figure S1).

### Neurobehavioural Development

The time spent immobile during the swim test did not vary with treatment (Figure S2). Similarly none of the variables assessed in the open field test varied with treatment (Figure S3). However, there was a significant difference in urine puddles produced in the open field test with CPS offspring urinating 80% less often than controls during the testing period (z = 2.3, P<0.05).

## Discussion

To gain an understanding of the potential impact of shift work during pregnancy on the long term health of the offspring, pregnant rats were subjected to chronic phase shifting of the photoperiod throughout gestation. We have shown for the first time that offspring born following this procedure had increased adiposity, increased plasma insulin, impaired glucose tolerance and glucose stimulated insulin secretion and increased whole body insulin resistance. These changes occurred despite the lack of effect of the prior exposure to CPS on litter size, birth or weaning weights. Some of these responses were gender and age dependent. The body weight of females born to CPS exposed mothers was greater that those born to control rats from 40 weeks of age and at 12 months of age had increased adiposity, reduced glucose tolerance and insulin sensitivity. By contrast, males born to CPS rats did not differ from the controls in weight at any age, showed evidence of increased adiposity only at 3 months of age, and had normal glucose and insulin tolerance at all ages. However, CPS offspring had increased plasma insulin and leptin at 3 months of age (males) while for the females this was evident at 12 months of age.

To simulate shift work, in these studies a chronic phase shift schedule was utilised whereby the photoperiod was shifted by 12 hours twice a week. Tsai and colleagues [12] used a similar procedure and found that rats made repeated attempts to re-entrain their activity and temperature rhythms to the changing photoperiod. Consequently significant circadian dysynchronisation occurred, with a reduction in the amplitude of both temperature and heart rate rhythms. Others have shown disruption to clock and clock controlled gene rhythms in peripheral tissues after exposure to CPS [13]. We therefore expect that the pregnant dams subjected to the CPS protocol experienced significant circadian disturbance.

Pregnancy poses significant metabolic challenges for the mother; by late gestation, pregnant rats have increased insulin resistance [14] which is compensated by endocrine pancreatic hypertrophy [15] and increased insulin secretion in response to glucose load [16] allowing maintenance of appropriate plasma glucose and insulin levels. However, perturbations in these mechanisms can give rise to gestational diabetes which has severe long term consequences for the offspring. In humans this is characterised by macrosomia, impaired glucose tolerance and increased risk of adult obesity and diabetes [17], [18]. In rodents, models of gestational diabetes produce offspring with glucose intolerance, impaired insulin secretion, insulin resistance and increased adiposity [19], [20], [21]. Depending upon the model and the degree of diabetes induced, gestational diabetes in rats has been reported to cause either macrosomia [22], [23] or alternatively increased incidence of small for gestational age offspring [24], [25]. In our study, CPS during pregnancy did not alter birth weight, nor increase the incidence of small or large for gestational age offspring. However, we cannot exclude the possibility that the metabolic alterations observed in the adult offspring of CPS exposed mothers were a consequence of prenatal hyperglycaemia exposure following reduced glucose tolerance and insulin sensitivity of the mother. Further studies on the metabolic status of pregnant rats exposed to CPS are underway and will help to uncover the possible mechanisms involved in the effects on offspring reported here.

Another possible mechanism through which the chronic phase shifting could affect the developing fetus is via the maternal Hypothalamic Pituitary Adrenal (HPA) axis if the regimen is a prenatal stressor resulting in elevated maternal glucocorticoids. Prenatal stress has been reported to either decrease [26], [27], [28], increase [29], or have no effect on birth weight [30], [31], although varying methods of prenatal stress induction and statistical analyses of birth weight [32] may have confounded the results. Unlike synthetic glucocorticoid administration [33], [34], [35], there is less evidence that prenatal stress exposure alters metabolic homeostasis in the offspring. Prenatal restraint stress had no effect on either glucose homeostasis or insulin secretion of 4 month old rats [31]. However, analysis of much older rats (24 months) revealed hyperglycaemia and reduced glucose tolerance, but no effects on plasma insulin or glucose induced insulin secretion were observed [36]. Clearly further studies of the maternal HPA axis in response to CPS exposure are required to determine the contribution of prenatal stress to the metabolic alterations observed in the offspring.

It has been shown in both human and animal studies that prenatal stress influences neurobiology and behaviour of the adult offspring [9], [37]. For example, exposure of pregnant rats to a wide range of physical and psychological stressors can increase depressive and anxiety-like behaviours in the offspring [38], [39]. Our data demonstrates that offspring of dams exposed to CPS do not show either increased immobility in the forced swim test, nor reduced explorative activity in the open field test. This suggests that any stress experienced by the dams following exposure to the CPS schedule was of insufficient intensity to affect neurobehavioural development in the offspring.

In the current study gender-dependent metabolic responses to the CPS were observed such that females were more susceptible to the metabolic impact of maternal CPS exposure. Gender-specific responses to *in utero* programming of metabolic function have been reported for protein restriction [40], [41], caloric restriction [42] and glucocorticoid exposure [43]. Typically, however, it is the males rather than females that are more susceptible to prenatal insults. It is not clear why females were more susceptible to the effects of CPS, but males may have eventually gone on to develop reduced glucose tolerance and insulin sensitivity since plasma insulin and leptin were already elevated at 12 months of age. It could also be expected that the feeding of diabetogenic diets to offspring of CPS exposed mothers would accelerate the deterioration of metabolic homeostasis in both males and females, as could the increased metabolic demands of pregnancy.

The hyperleptinaemia observed in both males and females is indicative of altered adipocyte function and increased adiposity, however, increased fat deposit were not always associated with high leptin levels. For example, 12 month old males had a 2-fold increase in plasma leptin, but epigonadal or retroperitoneal fat pad weights were not altered. Dual energy X-ray absorptiometry (DEXA) for rats was not available to us for this study, but may have provided better insight into the sources of the elevated leptin. Nevertheless, the consistent observations of hyperleptinaemia and increased fat deposits in some cohorts of males and females suggest exposure to the CPS during pregnancy alters adipocyte numbers and/or function.

The rats were subjected to the CPS protocol from the day after conception, throughout gestation, and into the postnatal period until the pups were 1 week old. Thus we cannot determine from our experiment what stage of gestation, or postnatal development, the fetuses are most vulnerable. Pups born to dams exposed to the CPS did not differ in birthweight, and pre-weaning growth was not affected, suggesting lactation was not impaired. However, the timing of feeding may have been disturbed during the first week and contributed to the altered metabolic function. The effects of CPS on the pregnant dam and developing fetus/pups may be cumulative since there were 8 photoperiod shifts during the study. Nevertheless, experiments limiting exposure to the CPS protocol to early or late gestation or early postnatal life may allow us to determine the stages of development most susceptible to the insult.

Exposure to CPS during gestation did not alter core body temperature rhythmicity in either light/dark or constant darkness conditions in the adult offspring. Similarly, phase shifts induced by nocturnal light exposure were similar for both CPS and control offspring. We had hypothesised that exposure to the CPS protocol, and the significant circadian disruption that this creates, would significantly impact on the developing fetal SCN with consequences for rhythmicity as adults. This conjecture was supported by evidence demonstrating prenatal insults such as protein restriction [44], [45], stress [46], hypoxia [47] and drug exposure [48] can disrupt aspects of circadian physiology in the offspring. Furthermore, perinatal protein restriction has recently been demonstrated to affect the circadian transcription of genes regulating food intake and metabolism of the adult offspring [45]. Coincidentally, poor pregnancy outcomes such as prematurity or small size at birth, which predispose an individual to adult metabolic disorders, also increase the incidence of distorted melatonin production [49]. Nevertheless, our data suggests that while repeated photoperiod phase shifts experienced throughout gestation alter the metabolic health of the offspring, SCN development and function are not affected.

In conclusion, these experiments have demonstrated for the first time the metabolic consequences for offspring of pregnant rats exposed to chronic phase shifts during gestation. The circadian rhythm disruption that is produced by the protocol can be likened to the circadian disruption experienced by human shift workers. Therefore, an analysis of the impact of shift work exposure during pregnancy on subsequent human adult metabolic health is warranted. In Australia for example, 16% of working women work shifts, primarily in the areas of accommodation and food services, and health care and social assistance [50]. While only a small percentage of these women may be pregnant at any one time, over the course of their working life it is expected that a significant proportion would have been pregnant at some stage. This suggests that potentially a large and increasing proportion of the population may have been exposed to shift work *in utero*, which in turn may contribute to the increasing incidence of obesity and related metabolic disorders in western society. It is therefore imperative that epidemiological studies are conducted to assess not only the impact of shift work on pregnancy outcomes, but also the health of the offspring into adulthood. This may then form the basis for changes in the scheduling of work periods for working pregnant women. Unlike most causes of chronic health disorders, reducing the risk associated with shift work during pregnancy should be achievable.

## Supporting Information

Figure S1 **Circadian rhythmicity of core body temperature in CPS and control offspring.** Core body temperature was recorded in 12L:12D (A) and constant darkness conditions (B) by iButtons® inserted into the peritoneal cavity of male CPS (grey) and control (black) offspring. Phase shifts in core body temperature offsets were calculated following light pulses (300 lux, 15 minutes) given at 2300h or 0500h (C, • control 2300h pulse, ○ CPS 2300h pulse, ▪ control 0500h pulse, □ CPS 0500h pulse).
(TIF)

Figure S2 **Behavioural despair in CPS and control offspring.** Time spent immobile during 5 minutes of testing in the forced swim test. The data are the mean ± SEM (n = 10 /treatment), CPS (□) control (•).
(TIF)

Figure S3 **Anxiety-like behaviours in CPS and control offspring.** Number of squares crossed, time taken to leave the initial square, number of outer squares crossed, % outer squares crossed, and urine puddles and faecal boli produced during the 5 minutes of testing in the open field test. The data are the mean ± SEM (n = 10 /treatment), *P<0.05. CPS (□) control (•).
(TIF)
