# Supplementary figures and images for: Chronic Phase Shifts of the Photoperiod throughout Pregnancy Programs Glucose Intolerance and Insulin Resistance in the Rat
Source: PLoS One. 2011 Apr 6;6(4):e18504. doi: 10.1371/journal.pone.0018504 (PMC3071829; doi:10.1371/journal.pone.0018504)

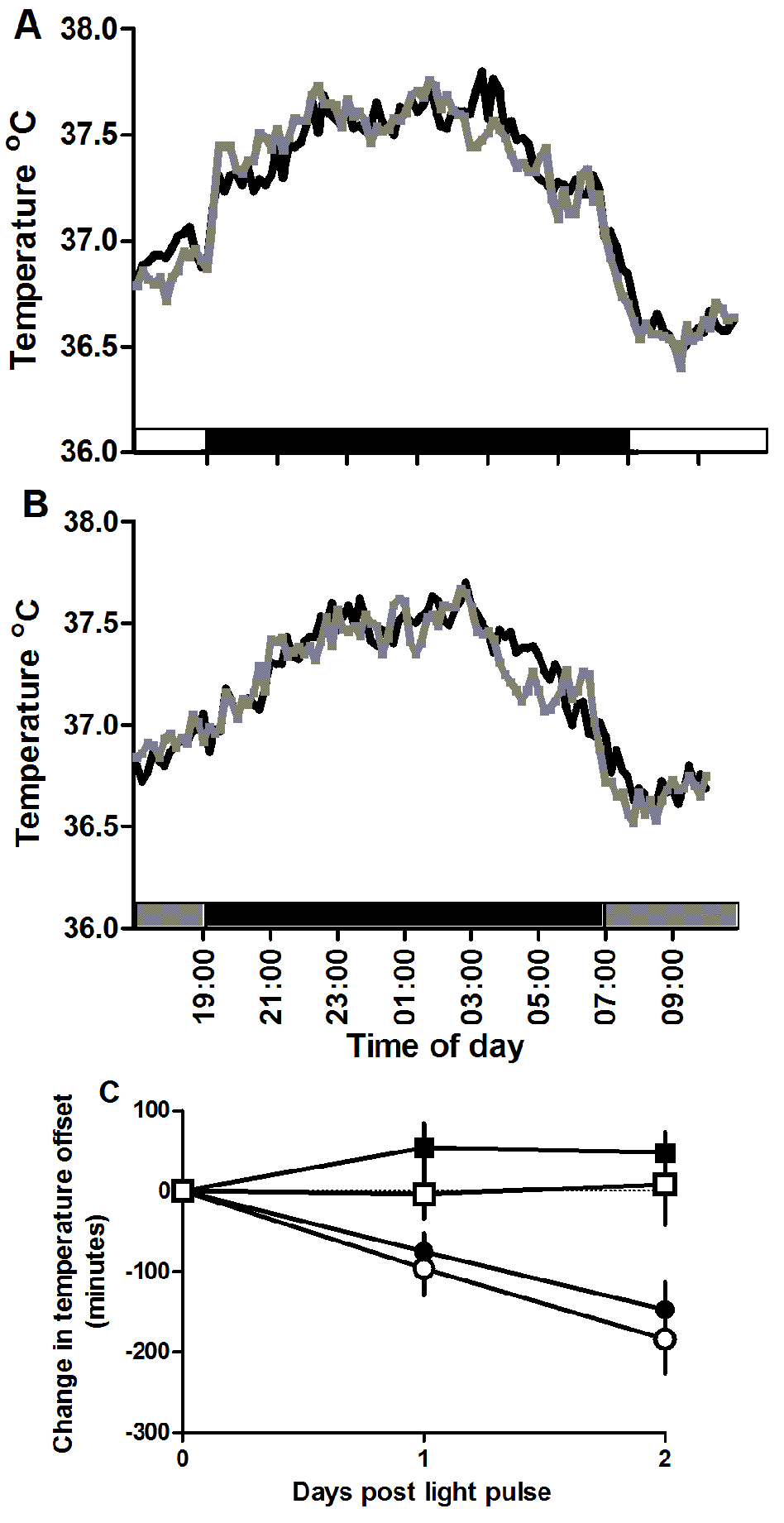

Supplement: Figure S1 — Circadian rhythmicity of core body temperature in CPS and control offspring. Core body temperature was recorded in 12L:12D (A) and constant darkness conditions (B) by iButtons® inserted into the peritoneal cavity of male CPS (grey) and control (black) offspring. Phase shifts in core body temperature offsets were calculated following light pulses (300 lux, 15 minutes) given at 2300h or 0500h (C, • control 2300h pulse, ○ CPS 2300h pulse, ▪ control 0500h pulse, □ CPS 0500h pulse). (TIF) [file pone.0018504.s001.tif]

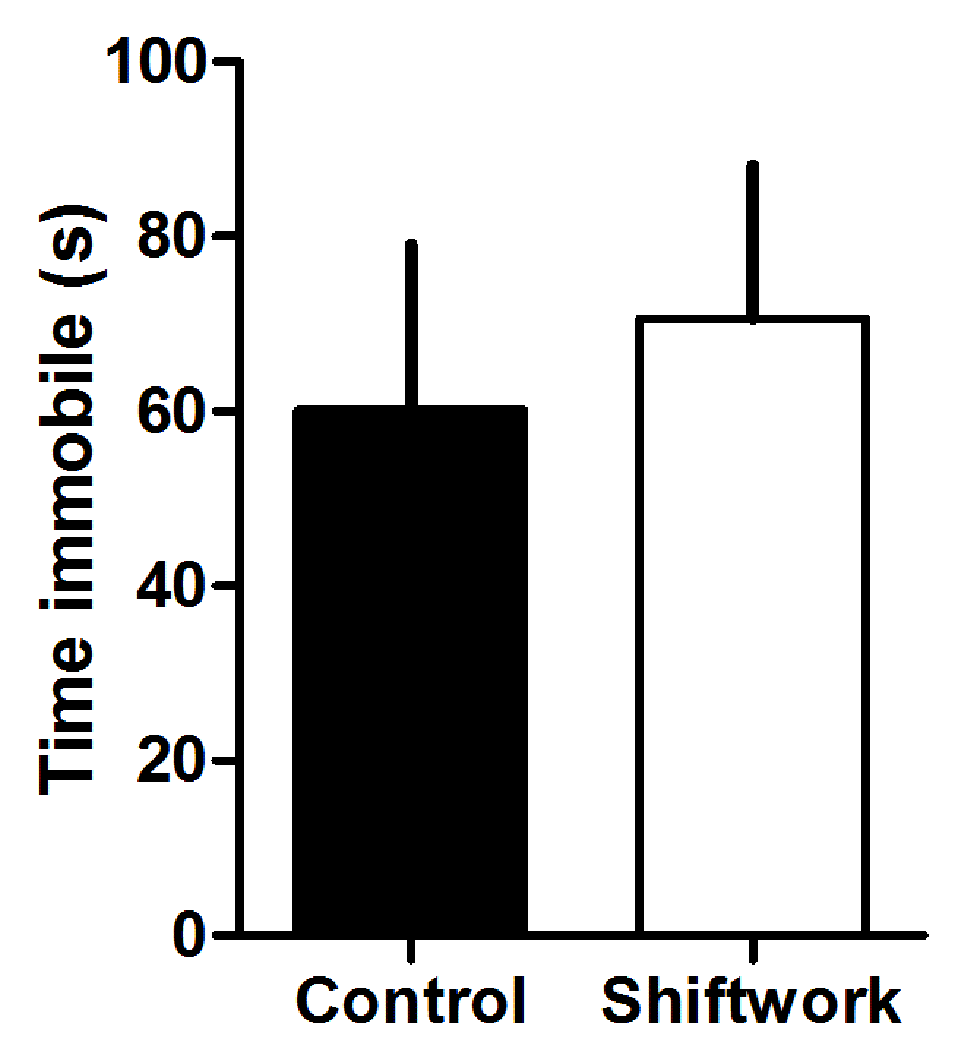

Supplement: Figure S2 — Behavioural despair in CPS and control offspring. Time spent immobile during 5 minutes of testing in the forced swim test. The data are the mean ± SEM (n = 10 /treatment), CPS (□) control (•). (TIF) [file pone.0018504.s002.tif]

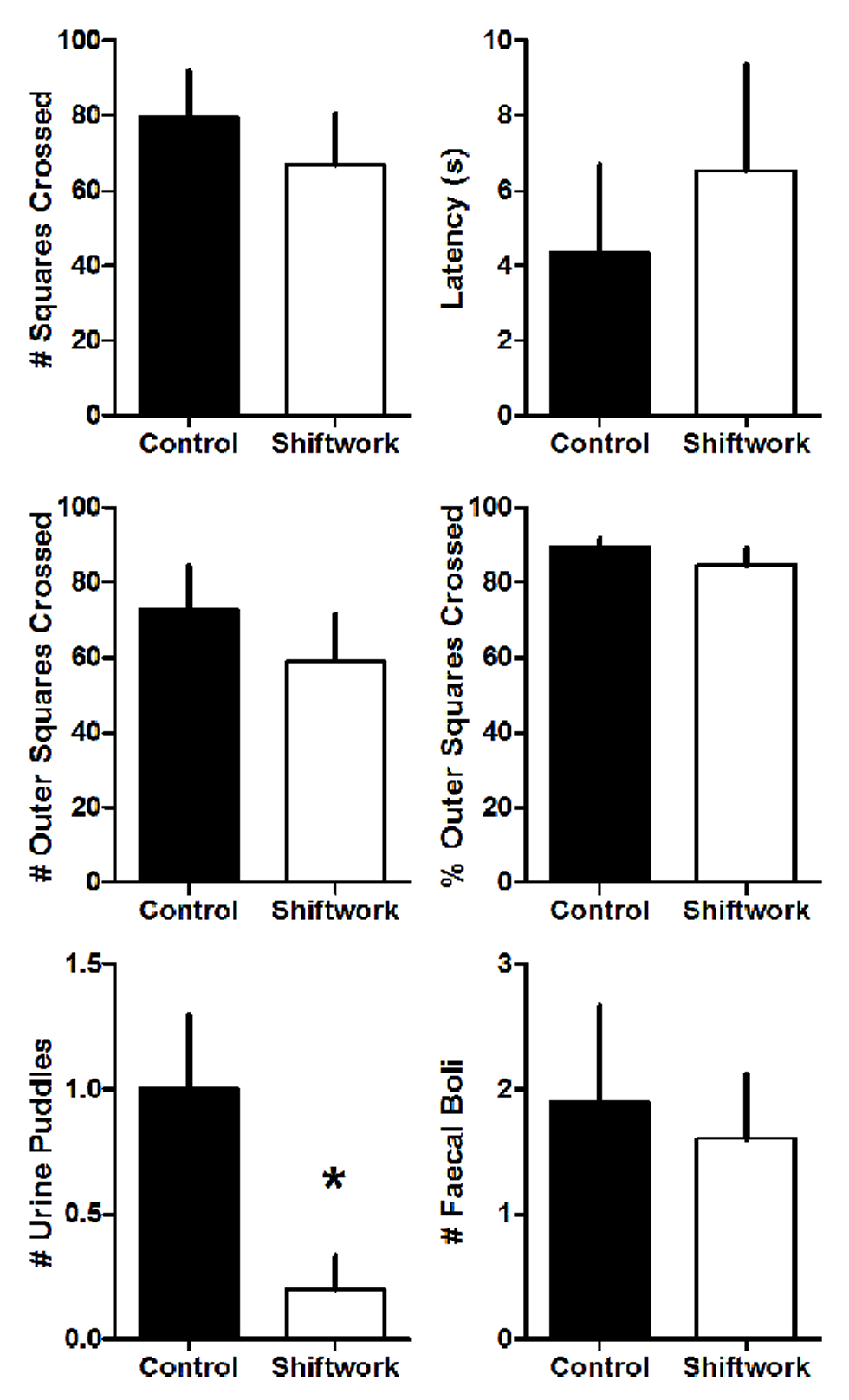

Supplement: Figure S3 — Anxiety-like behaviours in CPS and control offspring. Number of squares crossed, time taken to leave the initial square, number of outer squares crossed, % outer squares crossed, and urine puddles and faecal boli produced during the 5 minutes of testing in the open field test. The data are the mean ± SEM (n = 10 /treatment), *P<0.05. CPS (□) control (•). (TIF) [file pone.0018504.s003.tif]
